# Widespread promoter methylation of synaptic plasticity genes in long-term potentiation in the adult brain in vivo

**DOI:** 10.1186/s12864-017-3621-x

**Published:** 2017-03-23

**Authors:** Jesper L. V. Maag, Dominik C. Kaczorowski, Debabrata Panja, Timothy J. Peters, Clive R. Bramham, Karin Wibrand, Marcel E. Dinger

**Affiliations:** 1 Division of Genomics and Epigenetics, Garvan Institute of Medical Research, Sydney, Australia; 2 St Vincent’s Clinical School, Faculty of Medicine, University of New South Wales, 370 Victoria Street, Darlinghurst, Sydney, NSW 2010 Australia; 3 Department of Biomedicine and K.G. Jebsen Centre for Neuropsychiatric Disorders, University of Bergen, Bergen, Norway

**Keywords:** Long-term potentiation, LTP, DNA-methylation, MeDIP, Synaptic plasticity, Epigenetics, Neuroepigenetics

## Abstract

**Background:**

DNA methylation is a key modulator of gene expression in mammalian development and cellular differentiation, including neurons. To date, the role of DNA modifications in long-term potentiation (LTP) has not been explored.

**Results:**

To investigate the occurrence of DNA methylation changes in LTP, we undertook the first detailed study to describe the methylation status of all known LTP-associated genes during LTP induction in the dentate gyrus of live rats. Using a methylated DNA immunoprecipitation (MeDIP)-array, together with previously published matched RNA-seq and public histone modification data, we discover widespread changes in methylation status of LTP-genes. We further show that the expression of many LTP-genes is correlated with their methylation status. We show that these correlated genes are enriched for RNA-processing, active histone marks, and specific transcription factors. These data reveal that the synaptic activity-evoked methylation changes correlates with pre-existing activation of the chromatin landscape. Finally, we show that methylation of Brain-derived neurotrophic factor (Bdnf) CpG-islands correlates with isoform switching from transcripts containing exon IV to exon I.

**Conclusions:**

Together, these data provide the first evidence of widespread regulation of methylation status in LTP-associated genes.

**Electronic supplementary material:**

The online version of this article (doi:10.1186/s12864-017-3621-x) contains supplementary material, which is available to authorized users.

## Background

Long-term potentiation (LTP), induced by high-frequency stimulation (HFS), is a model for synaptic plasticity recapitulating the neuronal response to learning [1]. Several studies have shown early changes in kinase activity, transcription factors, mRNA, miRNA, and lncRNAs in response to LTP [2–7]. However, the involvement of the epigenome in LTP has largely been overlooked [8].

Methylation of DNA, which involves the addition of a methyl group to cytosine (5mC, 5-methylcytosine), is an important regulating process in promoters and CpG-islands where it can modulate gene expression (reviewed in [9]). Although increased DNA methylation often leads to decreased expression, the relationship between methylation and expression is not fully understood [10].

DNA methylation has been shown to be required for mammalian development [11], and for neuronal cell differentiation [12, 13]. Interestingly, studies have also shown that DNA methylation plays a role in the adult brain. For example, knockout of *de novo* DNA methylation proteins, such as Dnmt1 and Dnmt3a, led to learning deficits in mice [14]. Changes to DNA methylation in *Bdnf*, an important synaptic plasticity gene, have been shown to regulate *Bdnf* expression [15], and remodel chromatin after *Bdnf* stimulation [16], a stimulus known to evoke synaptic plasticity. DNA methylation is also a feature of contextual learning [17] and electroconvulsive stimulation [18] where CpG-islands were identified as having changed methylation status, revealing a potential role for methylation/demethylation in post-synaptic neurons.

Although DNA-methylation has been known to be involved in LTP [19], to date, no study has attempted to observe the scale of methylation changes in LTP. In a previous study of RNA expression, we observed robust increases in expression of *Tet3* [5], an enzyme involved in DNA demethylation. Tet3 oxidises 5mC to 5hmC, which is the first step in the removal of the methyl group from 5mC [20, 21] and is required in homeostatic plasticity [22]. Whereas all Tet-proteins catalyse the same reaction [21], an expression analysis showing an increase in only *Tet3* suggests it is the primary contributor to the oxidation [20]. Taking these observations together, we set out to investigate the role of methylation in LTP.

Using a rat model, we present the first study exploring changes in DNA methylation in LTP-associated genes in response to HFS. An analysis of methylation state in relation to gene expression and chromatin remodeling suggests that these genes are already primed for transcription by active chromatin. Together, these data suggest an active regulatory role for DNA methylation and histone modification in synaptic plasticity and memory formation.

## Results

### Long-term potentiation induction through HFS

The long-term potentiation was induced as described previously [5]. Briefly, the left *Dentate gyrus* (DG) in the hippocampus of urethane-anesthetised rats were subjected to high-frequency stimulation (HFS), using a well-established pattern paradigm, of medial perforant path input to granule cells of the DG. This stimulation resulted in a sharp and lasting increase of the perforant path-evoked field excitatory post-synaptic potential. Matched RNA-seq data was downloaded from ArrayExpress (E-MTAB-3375) and analysed to find 1,110 unique differential expressed genes using a stringent expression threshold described in Methods. While the RNA-seq study utilised the matched contralateral unstimulated DG, this study used baseline test-pulse stimulation as the control group and compared the methylation changes to 3 different time points after HFS. These time points were 30 min, 2 h, and 5 h post-HFS. Only the 6 stimulated samples were matched between the methylation array and the previous RNA-seq study. The expression data from the unstimulated naïve control group from the RNA-seq study was used as a surrogate to investigate the relation between expression and methylation in the base-line test stimuli group.

### Design of methylation array of LTP associated gene promoters

**Fig. 1:**
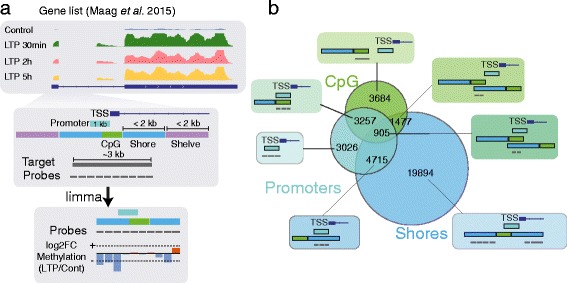
Design of MeDIP-array for previously LTP associated genes. **a** Genes identified as differentially expressed in Maag et al. 2015, with an absolute logFC ≥ 0.9 and FDR ≤ 0.05, were targeted in this array. Briefly, probes were designed around an area of 3 kb of the TSS and CpG islands associated with these genes. The intensity of each probe was calculated using limma. In total, 38,119 probes were created for this experiment. **b** Each probe was collapsed into their specific group based on their location near their associated gene. Probes could be located in promoters, CpG-islands, shore, or a combination of all the previous elements. The majority of probes fell into the shores region

To examine the involvement of methylation changes in LTP, we designed a MeDIP-array based on the differentially expressed genes described above. Briefly, probes were designed for the promoter and gene-associated CpG-islands and CpG-shores for each differentially expressed gene, covering a domain ±3 kb around the transcription start site (TSS) (Fig. 1a). The design led to each probe hybridising to a specific region characterised by a CpG-island, CpG-shore, promoter, or a combination of the above (Fig. 1b). The resulting merged domains consisted of 5612 unique regions surrounding the promoter of the LTP-associated genes previously described.

### Methylation array reveals early changes in methylation around LTP genes

To investigate changes in methylation status resulting from HFS, we used limma [23] to determine the differential methylation status of probes (DMP) or collapsed regions (DMR) (see Methods) between HFS stimulated samples (30 min, 2 h, and 5 h) compared to test-pulse stimulation controls.

**Fig. 2:**
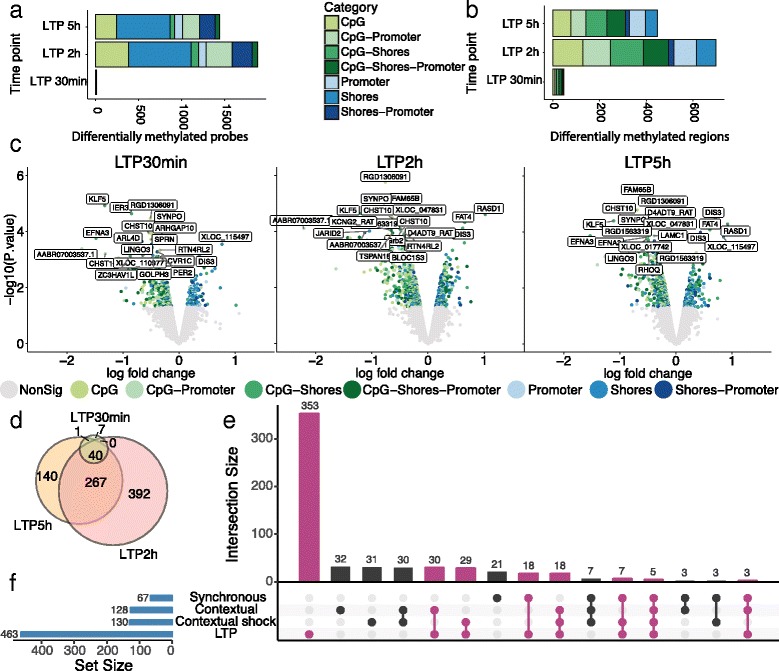
Differential methylation analysis reveals the majority of regions being downregulated in response to high-frequency stimulation. Number of differentially methylated (DM) (**a**) probes and (**b**) regions when comparing the stimulated samples with the controls. **c** Volcano plot showing the log2 fold change value plotted against the *p*-value for all regions per time point compared to control. Non-significantly methylated regions are coloured grey. The colours represent the different locations for each probe and region. The name of the top 20 differentially methylated regions for each time point are shown. **d** Venn diagram of DMRs from (**c**). **e** Comparison of methylation changes between LTP, single electroconvulsive stimulation [18], and contextual fear with our without shock [17]. **f** Total numbers of differentially methylated array-specific genes in each study. Only genes present in our methylation array were included, and all different methylated regions were collapsed into corresponding unique gene names. Bars in purple are overlaps with our LTP results

Few changes (11) in probe intensity were observed after 30 min while 1919 and 1474 probes were differentially methylated (FDR ≤ 0.25) at 2 and 5 h, respectively (Fig. 2a). Interestingly, when investigating differentially methylated regions, 48 regions displayed methylation changes after 30 min while 699 and 448 regions showed changes after 2 and 5 h (Fig. 2b).

To explore the direction of methylation changes, probes and regions were divided into their specific locations revealing an overall mean loss of methylation in CpG-associated regions, while methylation in promoters and shores increased (Additional file 1: Figure S1a, b). Overall, the majority (88% at 30 min, 71% at 2 h, and 75% at 5 h) of DMRs lose part of their methylation with stimulation (Fig. 2c; p-value ≤ 0.05). For example, immediate early responder gene *Arl4d* [24]*,* is highly differentially expressed at 30 min, displays a loss of methylation in the promoter region. The differentially methylated regions can be found in Additional file 2: Table S1.

The loss of methylation after HFS is consistent with our previous study [5] showing upregulation of the majority of genes after HFS, as methylation is mainly known to negatively correlate with gene expression. However, the loss of methylation at 30 min is not explained by the increase of *Tet3* expression, which increases only 2 h after HFS (Additional file 3: Figure S2). Moreover, the increase in DNA-methylation could be attributed to the increased expression of Dnmt3a (Additional file 3: Figure S2), which has previously been shown to be required for memory formation [25].

Comparison of DMRs between time points shows a similar trend in changes to that observed for differentially expressed genes (Fig. 2d) [5], where the majority of changes are similar between 2 and 5 h.

We further investigated the specificity of the methylation response in LTP by comparing the expression of our studied genes with changes reported in response to a single electroconvulsive stimulus [18], and contextual fear conditioning [17]. Although our studied genes only made up a subset of genes having differential methylation in their proximity in the other studies, 24% (110/463 genes) of our differentially methylated genes displayed methylation changes in response to other stimuli (Fig. 2e, f).

Taken together, these data suggest that changes to methylation occur in LTP, revealing a high specificity of methylation and demethylation events post-HFS.

### Methylation changes correlates with gene expression, and correlated genes are enriched for RNA processing and binding

Whereas site-specific differential methylation reveals changes at a certain moment, this analysis misses trends observed over multiple time points. Furthermore, it is important to investigate the relationship between methylation and expression over time. Previous studies have shown that the loss of methylation correlates with increases in gene expression, although the direction of correlation can be uncertain [10].

**Fig. 3:**
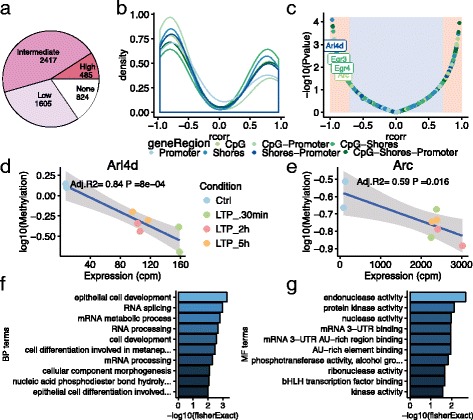
Correlation between methylation and expression status shows enrichment for RNA-splicing, kinase, and 3-UTR binding. **a** Effect of correlation between regions and their corresponding genes (**b**) Density plot of highly correlated genes (*r* ≥ 0.7 and *p*-value ≤ 0.05) (**c**) association between correlation value and *p*-value. Examples of LTP-associated genes (**d**) Arl4d and (**e**) Arc and the correlation between expression and methylation. Enrichment of (**f**) biological processes and (**g**) molecular functions of Methylation/expression correlated genes against non-correlated genes in the array

To investigate how methylation changes drive gene expression in LTP, we used the matched RNA-seq (E-MTAB-3375) counts per million reads (cpm), and the resulting logFC changes between each time point, from our previous study [5]. Since our methylation study utilised a baseline test stimuli as control instead of the contralateral DG as in the RNA-seq study, we chose the unstimulated naïve rat control as expression control. Comparison between the logFC between expression and methylation reveals that most genes/regions are negatively correlated (Additional file 4: Figure S3a). In contrast, when correlating the normalised expression with the normalised methylation values, we observed 825 regions that showed no correlation, 1605 regions that showed low correlation, 2417 regions that showed intermediate correlation, and 485 regions that showed high correlation between methylation changes and expression changes (Additional file 4: Figure S3a). When also accounting for *p*-value < 0.05, we observe only 455 regions showing high correlation with their corresponding gene, the majority (274/455, or 60%) of which are negatively correlated (Fig. 3b and c). Two genes that showed high negative correlation between expression and methylation are *Arl4d* (Fig. 3d) and *Arc* (Fig. 3e), both immediate early responder genes (IEGs), which show increased expression after only 30 min post-HFS. In *Arc*, the promoter associated CpG-island has previously been described to have altered methylation changes in response to spatial behavioural tasks in the hippocampus [26]. Moreover, several other genes show high linear correlation between their methylation and expression (Additional file 5: Figure S4), such as *Egr2-4* (Additional file 5: Figure S4a), *Etv5*, *Zswim5* and *Ube2g1* (Additional file 5: Figure S4b), and genes with later stage expression such as *Stam*, *Bhlhe40*, and *Jak2* (Additional file 5: Figure S4c). Highly correlated (absolute(r) ≥ 0.8) genes showed enrichment for RNA-processing (Fig. 3f) and 3′-UTR binding (Fig. 3g) compared to the rest of the genes in the array.

In summary, the majority of observed methylation changes post-HFS correlate with gene expression negatively whereas loss of methylation correlates with increased gene expression.

### Expression/methylation-correlated genes are enriched for motifs involved in brain function and development

To further understand the basis for the inconsistency between promoter methylation and gene expression, we examined the difference in the characteristics between LTP-associated genes where promoter methylation and expression were correlated and those that were not. We hypothesised that a decrease in methylation around the promoter could facilitate transcription factor binding leading to increased gene expression.

**Fig. 4:**
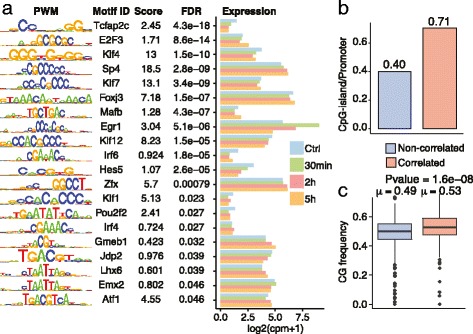
Motif search in promoter region of correlated genes show enrichment for CG-rich motifs. **a** Motifs enriched in significantly methylation/expression correlated 1 kb upstream of the TSS compared to non-correlated genes. **b** Average number of CpG-islands present in 1 kb upstream from the promoter in both groups compared to number of promoters in each groups. **c** Boxplots to the right show the GC frequency of the significantly and non-significantly correlated genes. μ represents the mean for each group. *p*-value was calculated using the Students’ *t*-test

In support with of this hypothesis, a motif search of expressed transcription factors between correlated and non-correlated gene promoters led to the discovery of enriched (FDR ≤ 0.05) CG-rich motifs 1 kb upstream of TSS in correlated genes corresponding to *Tcfap2c*, *E2F3*, *Klf4*, *SP4*, and *Egr1* (Fig. 4a). We observed a higher fraction of CpG-islands, and a small but statistically significant (μ = 0.53 vs. 0.49 correlated vs. non-correlated, *p*-value = 1.6e^-08^) increase of GC-content in our correlated vs. non-correlated genes (Fig. 4b, c), potentially confounding our motif analysis. To correct for GC-content and CpG-islands in this analysis, we only compared motifs present in promoters overlapping a CpG-islands in our two groups (Additional file 6: Figure S5). Although, some of these motifs were no longer enriched after filtering for promoters overlapping a CpG-island, *Egr1* and *SP4* remained (Additional file 6: Figure S5a). These genes are involved in brain function as drivers in synaptic plasticity and as a regulator of AMPA receptor subunits, respectively [27, 28]. In support of these findings, after filtering the number of CpG-island per transcripts and the CG-frequency of the promoters were almost identical (Additional file 6: Figure S5b), although, a slight enrichment in CG-content in correlated genes still remained (Additional file 6: Figure S5c). To further investigate the bias in GC-content and our results, we investigated the relationship between GC-content in probes (Additional file 7: Figure S6a), regions (Additional file 7: Figure S6b), and the methylated regions correlation with gene expression (Additional file 7: Figure S6c). We observed no clear relationship between the GC-content and correlation between methylation and expression changes. This suggests that these motifs found in this analysis are not relics of the increased CpG-islands, but are important components of LTP, as exemplified by *SP4* and *Egr1*.

### Active chromatin marks show enrichment over expression/methylation-correlated genes

**Fig. 5:**
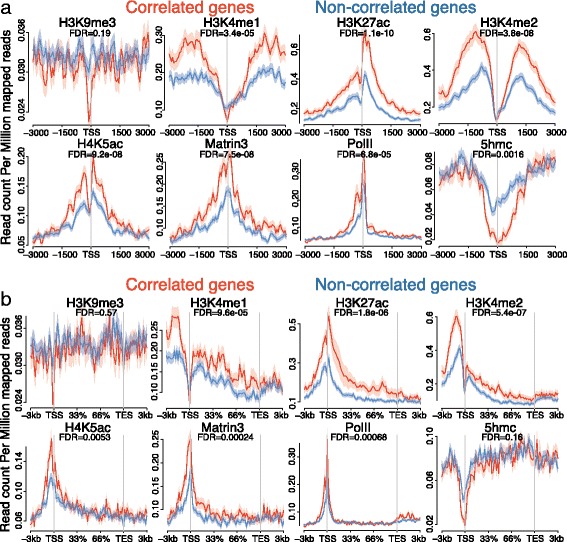
Chromatin marks show enrichment over correlated genes in normal rat brain. Public ChIP-seq control data sets were downloaded and analysed over the correlated and non-correlated genes with the respect of their (**a**) TSS ± 3 kb or (**b**) the whole gene body ± 3 kb from TSS and TES. H3K27ac, H2K4me2, H3K4me1, and Matrin 3 was obtained from a growth hormone (GH)- expressing rat pituitary cell line [49]), H3K9me3 from rat hippocampal tissue [50], PolII from rat neuronal cultures [51], 5hmc-capture from rat brainstem [52], and H4K5ac from rat dorsal striatum [53]. Error margin shows ± sem. FDR was calculated using Benjamini-Hochberg correction after using Wilcox-test

To further investigate the differences between the expression/methylation-correlated and non-correlated genes, we wanted to explore the chromatin state surrounding the genes in both groups. The chromatin landscape regulates gene expression through modifications of the histone tails. To address this question, we examined publically available datasets reflecting the normal adult chromatin state of rat brain (see Methods). Genes correlated with their methylation status had a higher enrichment of active histone marks, such as H3K4me1, H3K27ac, H3K4me2, and H4K5ac, around their TSS compared to non-correlated genes (Fig. 5a). Moreover, expression/methylation-correlated genes have enrichment of Matrin3 and Pol II, while they are depleted in 5hmc modifications, and H3K9me3 around the TSS (Fig. 5a). These enrichments are also present over the whole gene body (Fig. 5b), although the highest difference appears around the TSS, which drives the significance in H4K5ac, Matrin 3, and Pol II. Depletion of either H3K9me3 or 5hmc was not found when investigating the whole gene body (Fig. 5b).

To investigate any bias in our histone mark analysis, we compared the expression distribution between expression/methylation-correlated and non-correlated genes. We observe an already increased expression in the naïve rat dentate gyrus of the expression/methylation-correlated genes (Additional file 8: Figure S7) (Control; *p*-value = 0.014, mean_correlated_ = 21 RPKM, mean_noncorrelated_ = 14 RPKM). This could potentially explain the enrichment of active chromatin marks in the expression/methylation-correlated group in naïve brain.

This expression difference increases with stimulation and time (Additional file 8: Figure S7) (30 min: mean_correlated_ = 35 RPKM, mean_noncorrelated_ = 23 RPKM; 2 h: mean_correlated_ = 44 RPKM, mean_noncorrelated_ = 26 RPKM; 5 h: mean_correlated_ = 50 RPKM, mean_noncorrelated_ = 29 RPKM) showing that the correlated genes have a sharper expression increase than the non-correlated genes. Taken together, these data suggest that genes with expression correlated with their methylation status are already transcriptionally more active than non-correlated genes, have an enrichment of active chromatin marks, and have a higher proportion of enriched neuro-specific motifs in their promoters.

### CpG-island methylation correlates with the isoform switching observed in the Bdnf loci in LTP

**Fig. 6:**
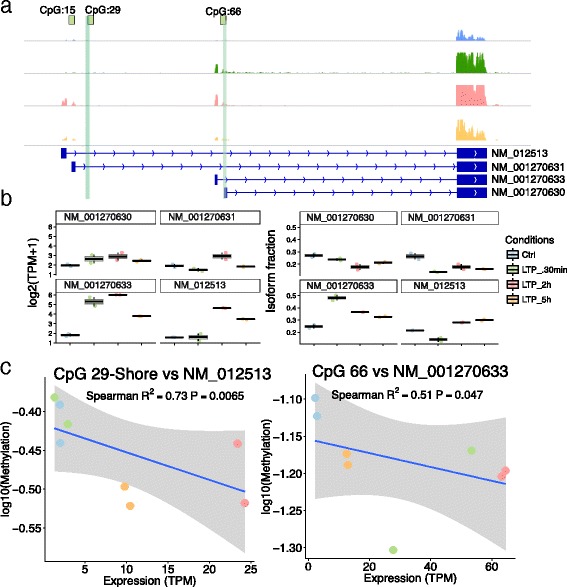
Time dependent differences in methylation status correlates with isoform switching of the Bdnf gene. **a** The Bdnf loci with the 4 major isoforms expressed in this study with associated CpG-islands. **b** The expression (*left*) and fraction (*right*) of the major Bdnf isoforms. **c** The correlation between methylation and expression of the NM_012513 (left) and NM_001270633 (*right*) isoforms of Bdnf and their associated CpG-island

Methylation at different promoters of the same gene can influence which isoform is expressed. To examine whether such a mechanism was employed in LTP, we focused on the expression of the *Bdnf* gene, which plays an important role in synaptic plasticity [16, 29]. Previously, demethylation of *Bdnf* has been shown to occur in the prefrontal cortex in response to LTP [30]. The existence of multiple *Bdnf* isoforms is well known [31], and switches in expressed *Bdnf* isoforms in fear conditioning is due to their differential methylation status [15]. The rat *Bdnf* loci consists of three CpG-islands, for which we designed probes along with corresponding CpG-shores (Fig. 6a). In our LTP model, 4 major isoforms of *Bdnf* are observed, with low expression of *Bdnf* in the control samples, one isoform lacking expression after 30 min while all isoforms are expressed after 2 h and 5 h post-HFS (Fig. 6b). These isoforms corresponds to exon I, exon II, exon IV, and exon VI [31]. Investigating these isoforms with their corresponding methylation status, we found two isoforms (exon I and exon IV) that show expression patterns consistent with their methylation status, suggesting that methylation may be employed here as a mechanism for isoform selection (Fig. 6c). The other two major isoforms (exon II and exon VI) expressed showed no correlation between methylation and expression, which suggests expression of these isoforms may be regulated by another mechanism.

## Discussion

The regulation of gene expression is a highly complex and tightly controlled process involving DNA methylation, transcription factors, and chromosome organisation.

The role of DNA methylation in LTP has so far been understudied. Here we present the first large-scale methylation study of temporal methylation changes in LTP and correlate the methylation status with matched RNA-seq data for LTP-associated genes. We find that only a few regions show rapid changes in DNA-methylation 30 min after LTP induction, while the majority of changes happen in the intermediate (2 h) to late term (5 h). We also show that genes whose expression correlates with their proximal methylation are enriched for RNA-binding and RNA-processing. Furthermore, we reveal specific transcription factor motifs are enriched in correlated gene promoters, independent of CpG-islands, and we show that these genes are enriched for active chromatin marks in steady state, indicating that these genes already show signs of transcription which are further altered by methylation changes.

The most common changes to promoter methylation is demethylation, supporting our previous observation of enhanced expression for the majority of LTP associated genes [5]. Although methylation levels are generally negatively correlated with expression, this is not always the case [10]. We observed the expression of genes that were both negatively and positively correlated with their methylation level. Although we do not observe any specific relationship between the location of the methylation changes and the correlation, changes may occur in regions not covered by our array. For example, the methylation of gene bodies can influence the expression levels [32]. Moreover, methylation changes in enhancer regions are associated with downstream changes to gene expression [33]. Further studies combining whole-genome bisulphite sequencing with ChIP-seq will be able to clarify the relationship.

Transcription factor binding sites (TFBS) in the promoters are important regulators of gene expression, and DNA methylation changes at TFBS can alter their binding [34], although it is not considered a general property for transcription factor binding [35]. Here we observe certain expressed transcription factors that are enriched in genes correlated with methylation changes. Specifically, we see enrichment of *Egr1*, which is highly expressed 30 min post-HFS. Moreover, we observe *SP4* and *Nfe2I2*, two transcription factors involved in expression of *AMPA* [27] and *NMDA* [36] type glutamate receptors, respectively. These transcription factors are known to have transcriptional control over important receptors involved in LTP. Here we show that their motifs are also enriched in the correlated genes, suggesting that they also control expression of many identified LTP-associated genes. We also observe enrichment of *Tcfap2c*, a transcription factor forming a complex with Cited2, which regulates *Cebpa* expression [37]. *Cited2* is a gene highly expressed 30 min post-HFS [5], and, together with *Cebpb* (differentially expressed after 2 and 5 h post HFS), is a neuronal activity dependent transcription factor [38]. However, the enrichment of *Tcfap2c* is possibly artifactual, as the enrichment diminished when selection was restricted to genes containing CpG-islands. Other TFs enriched in the promoter of correlated genes are *Foxj3*, *Mafb*, *Srebf1*, and a depletion of *Sox10*, all which have been linked to either brain development or differentiation [39–42].

Furthermore, we investigated the steady-state chromatin landscape between the correlated and non-correlated genes. Through this analysis, in correlated genes we observed enrichment of active chromatin marks, such as H3K4me1, H3K227ac, H3K4me2, H4K5ac and Matrin3 and PolII, and a slight decrease of inactive chromatin marks near the TSS, as shown by H3K9me3. The enrichment of active chromatin marks was also present over the whole gene body. This suggests that the genes responding to methylation changes are already primed for transcription by active chromatin.

Interestingly, the expression/methylation correlated genes showed a depletion of 5hmC compared to non-correlated genes, suggesting that the correlated genes are more methylated around their TSS than non-correlated genes. This supports our previous observation that Tet3 is responsible for the demethylation of 5mC in response to HFS.

We also provide evidence that CpG-island methylation around gene promoters correlates with the expression of specific *Bdnf* isoforms from transcripts containing exon IV to transcripts with exon I. However, this did not explain the expression changes of *Bdnf* isoforms containing exon II or exon VI.

Although we observe methylation changes to be a widespread phenomenon in LTP, the technique used, the limited sample size, and examined regions in this study suggests that the results provide an incomplete representation of the actual methylation changes in LTP. As our MeDIP-array relies on antibodies to capture the methylated regions, we are unable to get an absolute measurement of the methylation changes on a base resolution. Furthermore, the MeDIP-array can only distinguish large changes in methylation, suggesting we might miss more subtle changes. Instead, utilising whole-genome or targeted bisulphite sequencing should provide a more accurate representation, and provide detail of even miniscule difference, of the methylation changes observed in LTP.

As our study only covers the first few hours after HFS, we cannot say if the observed DNA methylation changes persists in the neurons or if the DNA methylation is more transient and goes back to normal later after HFS. Although we believe that the observed changes to DNA methylation in synaptic plasticity genes go back to normal after the required genes have been expressed, longer time points are needed to elucidate for how long these changes persists. Finally, further studies are needed to investigate how the methylation, hydroxymethylation, and chromatin landscape change in LTP in response to HFS.

## Conclusion

In conclusion, we show changes to the methylation landscape around synaptic plasticity genes during the first hours after LTP induction. Furthermore, we show that the histone landscape around correlated genes is already primed for transcription in steady-state, suggesting that an active chromatin landscape is required for DNA methylation to occur. This first large-scale study of DNA-methylation in LTP suggests a major role for epigenetic modifications in the activity-dependent control of gene expression underlying long-term synaptic plasticity in the adult mammalian brain.

## Methods

### Sample preparation

The animals and electrophysiology were the same as describe in previous study [5]. Briefly, after HFS to the dentate gyrus (DG) animals were decapitated at 30 min, 2 h, and 5 h, and the DG was micro-dissected and snap-frozen on dry ice. For the control group, a baseline test stimuli was applied instead of the HFS. This study used two animals per group. The field excitatory post-synaptic response for the different groups used in this study can be seen in Additional file 9: Figure S8a. After tissue homogenisation, the DNA was extracted using Allprep RNA/DNA isolation kit (Qiagen).

### Array design

Each differentially expressed gene from previous publication [5] with modified cut-off of absolute log2FC ≥ 0.9 and a FDR ≤ 0.05 was used in the array design. The FDR was obtained through the Benjamini Hochberg multiple testing corrections of *p*-values. Additionally, genes not annotated in Ensembl Rn4 69 release but previously characterised in LTP, such as Bdnf and Jarid2 were reanalysed using Refseq and included in the array along with the gene associated CpG-islands and shores. The coverage of each promoter with an associated CpG-island was set to ±1 kb from the TSS. Each CpG-island lacking gene was only covered at 0.5 kb upstream and downstream from the TSS, while the CpG-island coverage was extended with ±1.5 kb in each direction to accommodate for CpG-shores.

### Array preparation

The DNA methylation microarray was prepared following the Agilent microarray analysis of methylated DNA immunoprecipitation protocol V2.2. 5-methylcysteine antibody (A-1014-050) was ordered from Epigenetek. The hybridisation of samples for the array was conducted at the Ramaciotti Centre for Genomics at UNSW, where the arrays also were scanned.

### Array analysis

The arrays were read and analysed using limma [23]. Briefly, loess normalisation was used to normalise within arrays while quantile normalisation was used between the two arrays. Control probes were removed before analysis. Before quantile normalisation, the probe intensity of each sample was compared, and MDS plots of quantile normalised probes and regions were plotted (Additional file 9: Figure S8b-d). Each probe was given a unique location ID based on their respective region as in Fig. 1b. A contrast matrix was set up for differential methylation analysis between the control and stimulated samples, and probes were called differentially methylated if they had a *p*-value ≤ 0.05 and a FDR ≤ 0.25. For the region analysis, probes were averaged over their respective region as above in accordance with Fig. 1b. The regions were called differentially methylated regions (DMR) if they had a *p*-value ≤ 0.05 and a FDR ≤ 0.25.

### Comparing LTP differentially methylated genes with other models of neuronal stimulation

Gene lists with closest gene from differentially methylated areas were downloaded from published studies investigating the methylome changes of synchronous activation by electroconvulsive stimulation [18], and contextual fear condition with and without shock [17] from their respective supplementary material.

Since the downloaded gene lists contained mice genes, these were converted to rat gene names by first converting the mice gene names into orthologous rat ensembl ids using biomaRt. After that, the rat ensembl ids were converted to corresponding rat name using the ensembl 69 release. All differentially methylated regions were collapsed into their corresponding gene.

Only genes included in our array were investigated for the other stimuli. The resulting intersections were plotted using UpsetR [43].

### Correlation analysis

Gene expression values (cpm) was imported from previous study [5]. Each gene expression profile was correlated with its respective methylated region using the R-package Hmisc [44] with Pearson correlation. The effect of the correlation between gene expression and methylation was designated as following: r < 0.1 = no effect, 0.1 < r < 0.30 = small effect, 0.30 < r < 0.70 = intermediate effect, r > 0.70 = large effect. Only genes with a r > 0.70 and a P-value < 0.05 were called correlated in this study. Gene enrichment was done using TopGO on genes with a *r* ≥ 0.8. These highly correlated genes were compared to non-correlated genes in the array as a background. Both the biological and molecular function enrichment was conducted using the classic algorithm and fisher statistics from the TopGO package. For comparison of LogFC values between expression and methylation, logFC expression values were imported from Maag et al. 2015, and plotted against logFC methylation between each time point and control.

### Motif discovery and GC-content

Investigation for motifs present in correlated vs. non-correlated genes was done using PWMEnrich [45]. For the promoter region comparison region 1 kb upstream from the promoter were investigated for motifs. A background object was created using makePWMLognBackground for all *Mus musculus* motifs for the non-correlated promoters. For enhancers, the background was created the same way except for using enhancers neighbouring non-correlated genes as background. The promoter GC-content was calculated to investigate GC-bias, and the groups were compared using the student *t*-test. For the motif search, the correlated genes were enriched compared to the non-correlated genes using motifEnrichment with each specific background. Motifs were summarised using the groupReport function, and the top 20 motifs were plotted. Motif names were extracted from the R-package MotifDB. The *P*-values were adjusted using Benjamini & Hochberg adjustment.

### Probe and region GC-bias

All probes and regions were investigated for GC-bias between differentially methylated and non-differentially methylated probes or regions. The t-statistics from limma was plotted against the GC-content for each differential methylation comparison. Furthermore, the GC-content was also compared to the correlation between expression and methylation.

### Histone marks and transcription factors analysis over correlated genes

Briefly, the reads from public ChIP-seq control fastq files were aligned to the rat genome using Bowtie2 [46]. The aligned reads were then converted to bam, sorted, and indexed with Samtools [47]. Ngsplot [48] was used to calculate the average coverage of correlated and non-correlated genes over TSS ± 3 kb or the whole gene body ± 3 kb from TSS and TES. Significance of enrichment was calculated using the Wilcox test for log2 loci enrichment. The *p*-value for each histone mark was adjusted to account for multiple testing using Benjamini-Hochberg False discovery rate correction. H3K27ac, H2K4me2, H3K4me1, and Matrin 3 was obtained from a growth hormone (GH)- expressing rat pituitary cell line [49], H3K9me3 from rat hippocampal tissue [50], PolII from rat neuronal cultures [51], 5hmc-capture from rat brainstem [52] and H4K5ac from rat dorsal striatum [53].

### Expression differences between expression/methylation-correlated and non-correlated genes

To investigate the expression differences between the correlated and non-correlated genes, the RNA-seq count data was first converted to RPKM using the gene length from featureCount of the Rattus_norvegicus.RGSC3.4.69.gtf. This allowed for comparison between the two groups. Student’s *t*-test was used to observe significance between the correlated and non-correlated groups for each time point.

### Bdnf isoform switching by methylation

Due to Bdnf being absent from the Ensembl 69 Rn4, we counted reads with Rsem [54] using Refseq database for Rn4. Using the TPM from the 4 most prominent isoforms, we calculated the percentage of each isoform from the total gene expression. The TPM for each isoform was then correlated with the methylation of their closest CpG-island on the Bdnf loci to observe isoform-specific methylation events.

## Additional files

Additional file 1: Figure S1. Region specific log fold changes after stimulation. Violin plots showing the differentially methylated (a) probes and (b) regions for each time point compared to control. The number over each violin represents the mean logFC per probe or region. (PDF 1006 kb)

Additional file 2: Table S1. Differentially methylated regions for each time point. The differentially methylated regions discovered at 30 min, 2 h, and 5 h post-HFS. (XLSX 233 kb)

Additional file 3: Figure S2. Expression changes of methylation and de-methylation genes. Log2 fold changes in expression for Dnmt1, Dnmt3, Tet1, and Tet3 between stimulated and non-stimulated dentate gyrus. Data adapted from Maag et al. 2015. Control group is the naïve unstimulated samples from the RNA-seq study. Number on each bar shows the Benjamini Hochberg adjusted FDR values. (PDF 125 kb)

Additional file 4: Figure S3. Correlation between expression and methylation changes after HFS. Gene log fold changes of DE genes were imported from Maag et al. 2015. For each time point, all DE genes logFC were plotted against the logFC methylation after HFS vs. control. Each gene has multiple corresponding methylation regions based on figure 1b. Lines were plotted at ± 0.9 logFC expression and ± 0.1 logFC methylation. Each resulting quadrant shows the number of genes present. (PDF 118 kb)

Additional file 5: Figure S4. Examples of genes showing linear negative correlation between normalised expression and methylation. Genes showing linear correlation between methylation and expression through all time points from left to right, (a) *Egr2*, *Egr3*, *Egr4*. (b) *Etv5*, *Zswim4*, *Ube2g1*. (c) *Stam*, *Bhlhe40*, *Jak2*. (PDF 575 kb)

Additional file 6: Figure S5. Motif search in promoter region after removing all non-CpG-island overlapping promoters. (a) Motifs enriched in significantly methylation/expression correlated 1 kb upstream of the TSS compared to non-correlated genes in genes with CpG-islands present in the promoter region. (b) Number of CpG-islands present in 1 kb upstream from the promoter in both groups compared to number of promoters in each groups. (c) Boxplots to the right show the GC frequency of the significantly and non-significantly correlated genes. μ represents the mean for each group. *P*-value was calculated using the students’ *t*-test. (PDF 2830 kb)

Additional file 7: Figure S6. Investigation of GC-content in differentially methylated probes and regions reveals no bias between differential methylation and GC-content. GC-content of each (a) probe and each (b) region compared to the t-statistics from limma when comparing to control. (c) GC-content of each region compared to the correlation value when compared to gene expression. (PDF 2187 kb)

Additional file 8: Figure S7. Differences in expression levels between methylation/expression correlated and non-correlated genes. RPKM for correlated and non-correlated genes for each time-point. The lighter coloured boxplots show the non-correlated group while the darker coloured boxplots shows the methylation/expression correlated group. Statistics was calculated using student’s *t*-test. (PDF 134 kb)

Additional file 9: Figure S8. Group post-synaptic potential changes in response to HFS, and average array intensity and sample clustering. (a) The differences in the groups’ field excitatory post-synaptic potential after HFS. The data was taken from Maag *et* al. 2015, and included all 3 samples per group. The control group in this graph is the baseline test stimuli used as control in the present study (b) Distribution of probe intensity per samples before quantilie normalisation. MDS plot of (c) probes and (d) regions after quantile normalisation. (PDF 1131 kb)

## Abbreviations

5mC: 5-methylcytosine
DG: *Dentate Gyrus*
DMP: Differentially methylation probes
DMR: Differentially methylation regions, cpm, counts per million
HFS: High-frequency stimulation, TSS, transcription start site
IEG: Immediate early responder genes
LTP: Long-term potentiation
MeDIP: Methylated DNA immunoprecipitation
TFBS: Transcription factor binding sites
