# Supplementary material for: Widespread promoter methylation of synaptic plasticity genes in long-term potentiation in the adult brain in vivo
Source: BMC Genomics. 2017 Mar 23;18:250. doi: 10.1186/s12864-017-3621-x (PMC5364592; doi:10.1186/s12864-017-3621-x)
Supplement: Supplementary file 1 — Region specific log fold changes after stimulation. Violin plots showing the differentially methylated (a) probes and (b) regions for each time point compared to control. The number over each violin represents the mean logFC per probe or region. (PDF 1006 kb) [file 12864_2017_3621_MOESM1_ESM.pdf]

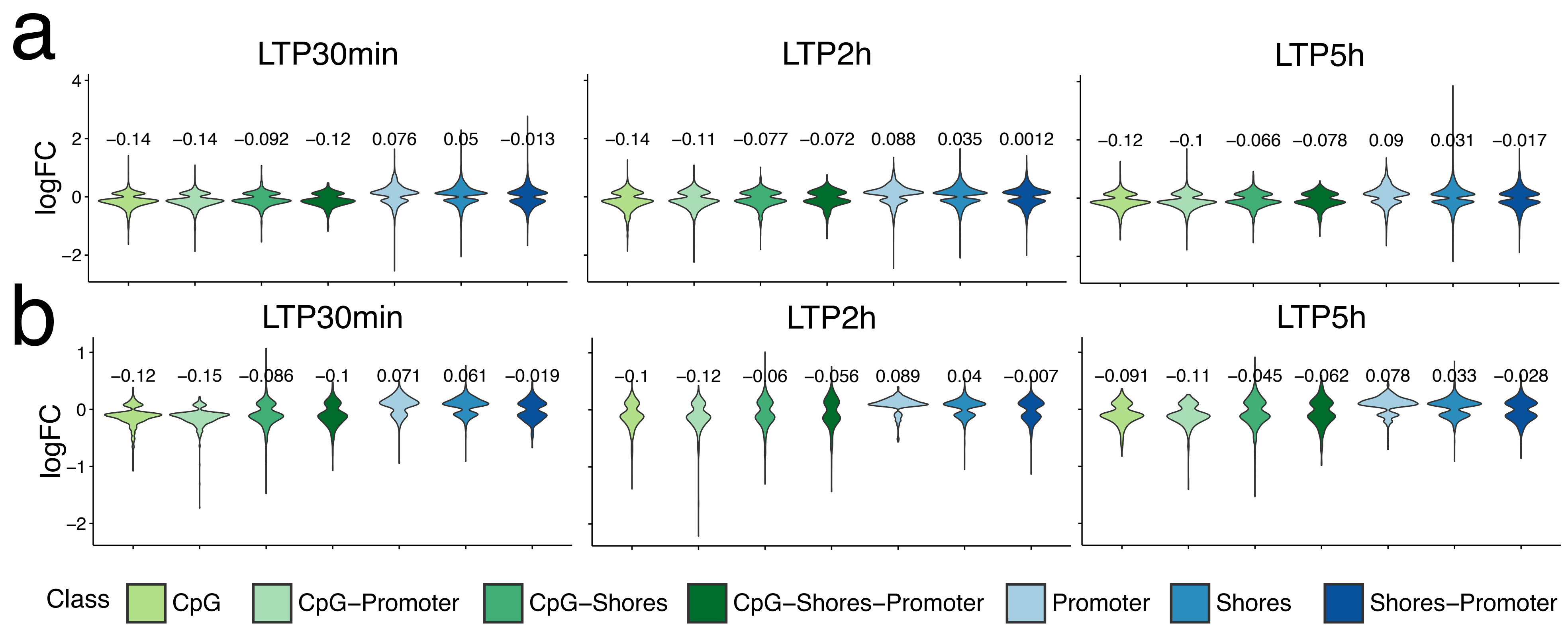
a
LTP30min
LTP2h
LTP5h
logFC
−0.14
−0.14
−0.092
−0.12
0.076
0.05
−0.013
−0.14
−0.11
−0.077
−0.072
0.088
0.035
0.0012
−0.12
−0.1
−0.066
−0.078
0.09
0.031
−0.017
b
LTP30min
LTP2h
LTP5h
logFC
−0.12
−0.15
−0.086
−0.1
0.071
0.061
−0.019
−0.1
−0.12
−0.06
−0.056
0.089
0.04
−0.007
−0.091
−0.11
−0.045
−0.062
0.078
0.033
−0.028
Class
CpG
CpG−Promoter
CpG−Shores
CpG−Shores−Promoter
Promoter
Shores
Shores−Promoter
