# Supplementary figures and images for: Widespread promoter methylation of synaptic plasticity genes in long-term potentiation in the adult brain in vivo
Source: BMC Genomics. 2017 Mar 23;18:250. doi: 10.1186/s12864-017-3621-x (PMC5364592; doi:10.1186/s12864-017-3621-x)

# Methylation genes

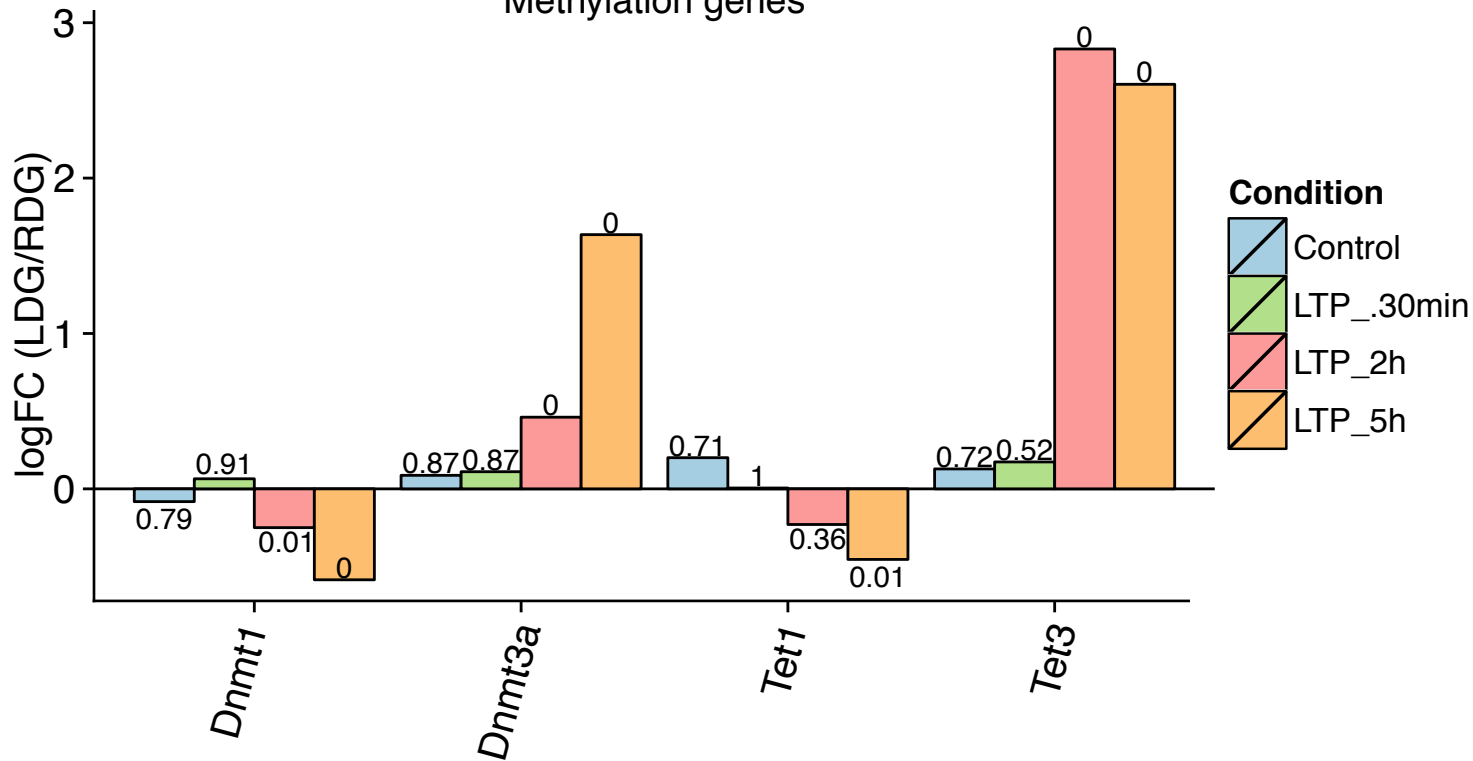

Supplement: Supplementary file 3 — Expression changes of methylation and de-methylation genes. Log2 fold changes in expression for Dnmt1, Dnmt3, Tet1, and Tet3 between stimulated and non-stimulated dentate gyrus. Data adapted from Maag et al. 2015. Control group is the naïve unstimulated samples from the RNA-seq study. Number on each bar shows the Benjamini Hochberg adjusted FDR values. (PDF 125 kb) [file 12864_2017_3621_MOESM3_ESM.pdf]

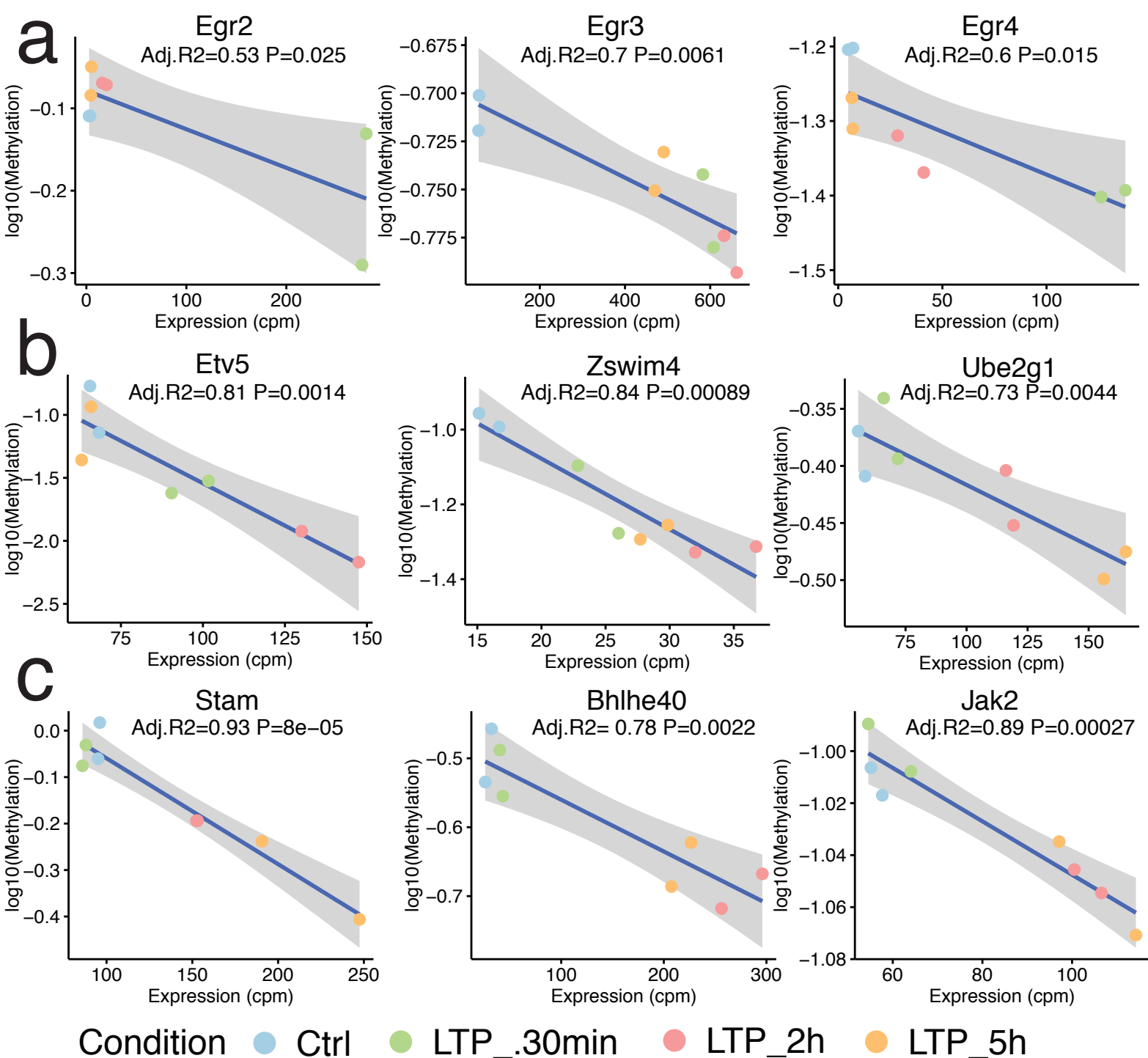

Supplement: Supplementary file 5 — Examples of genes showing linear negative correlation between normalised expression and methylation. Genes showing linear correlation between methylation and expression through all time points from left to right, (a) Egr2, Egr3, Egr4. (b) Etv5, Zswim4, Ube2g1. (c) Stam, Bhlhe40, Jak2. (PDF 575 kb) [file 12864_2017_3621_MOESM5_ESM.pdf]

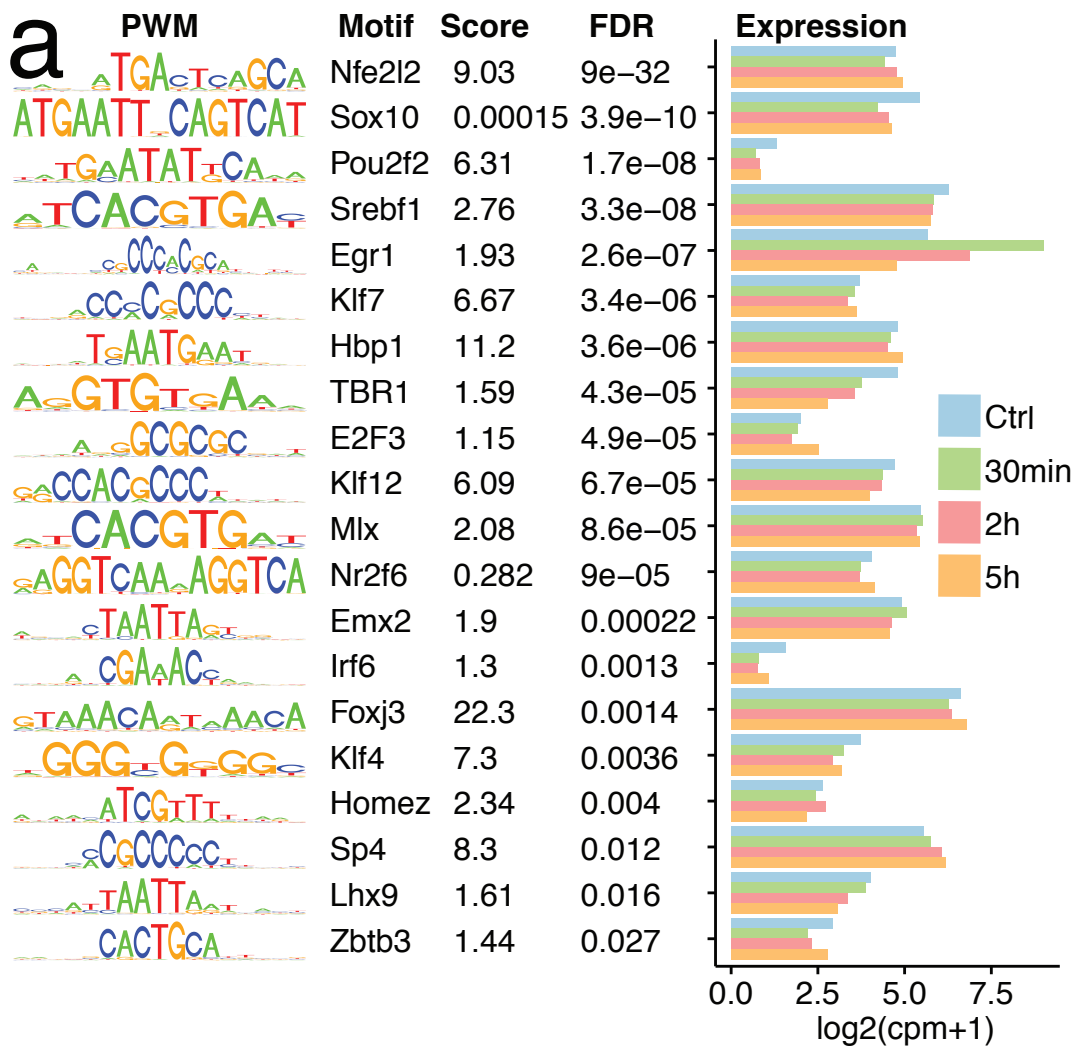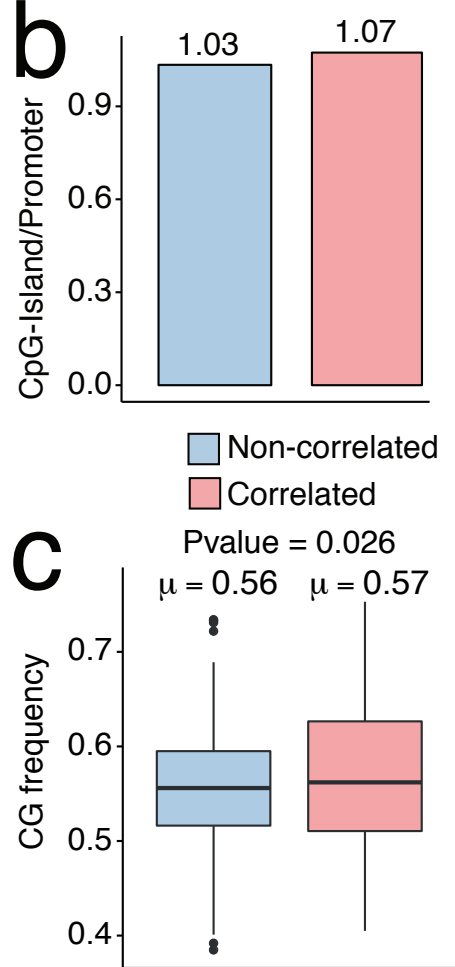

Supplement: Supplementary file 6 — Motif search in promoter region after removing all non-CpG-island overlapping promoters. (a) Motifs enriched in significantly methylation/expression correlated 1 kb upstream of the TSS compared to non-correlated genes in genes with CpG-islands present in the promoter region. (b) Number of CpG-islands present in 1 kb upstream from the promoter in both groups compared to number of promoters in each groups. (c) Boxplots to the right show the GC frequency of the significantly and non-significantly correlated genes. μ represents the mean for each group. P-value was calculated using the students’ t-test. (PDF 2830 kb) [file 12864_2017_3621_MOESM6_ESM.pdf]

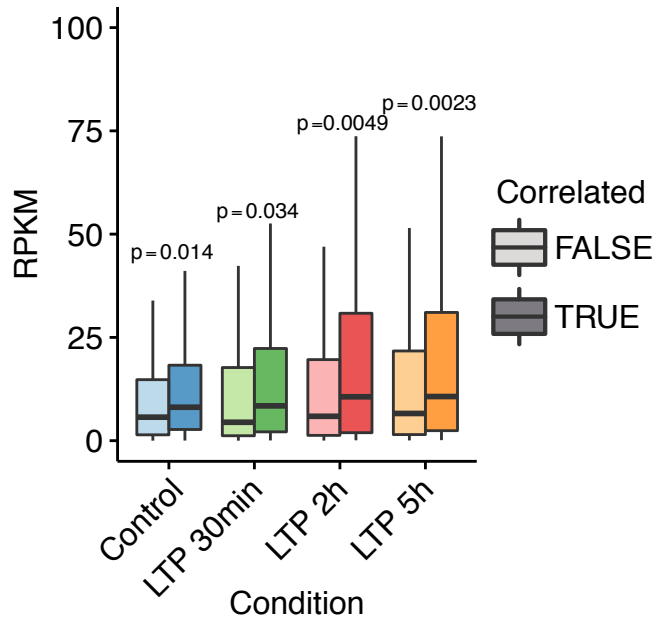

Supplement: Supplementary file 8 — Differences in expression levels between methylation/expression correlated and non-correlated genes. RPKM for correlated and non-correlated genes for each time-point. The lighter coloured boxplots show the non-correlated group while the darker coloured boxplots shows the methylation/expression correlated group. Statistics was calculated using student’s t-test. (PDF 134 kb) [file 12864_2017_3621_MOESM8_ESM.pdf]

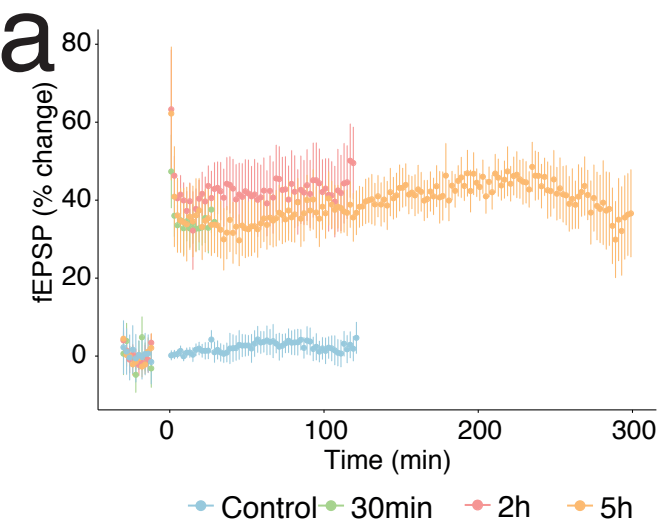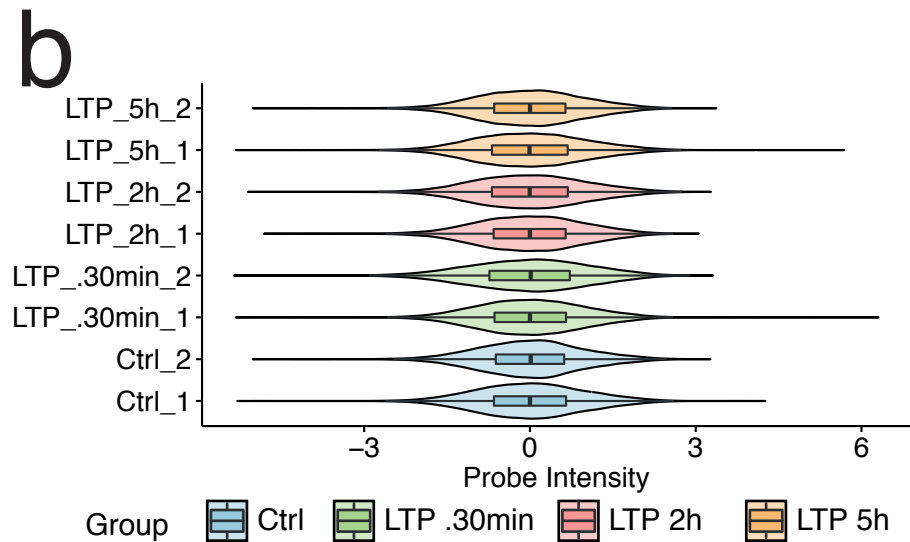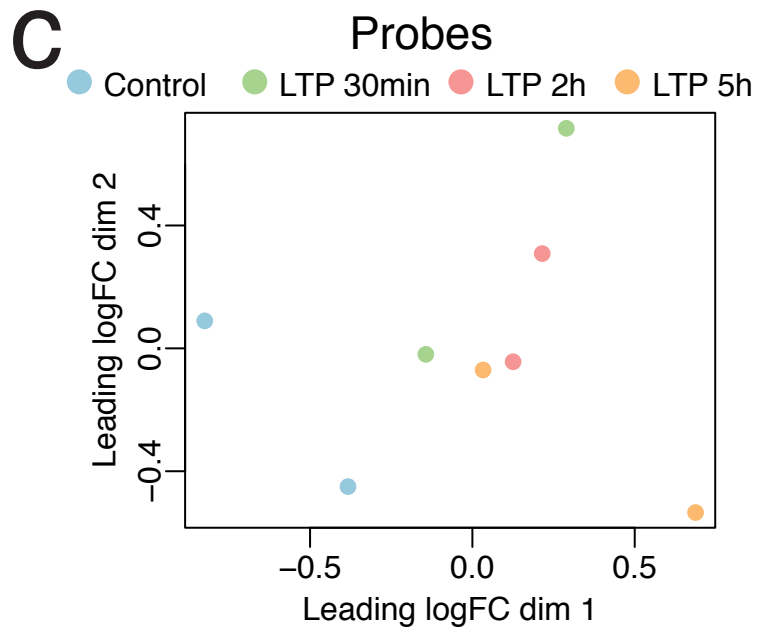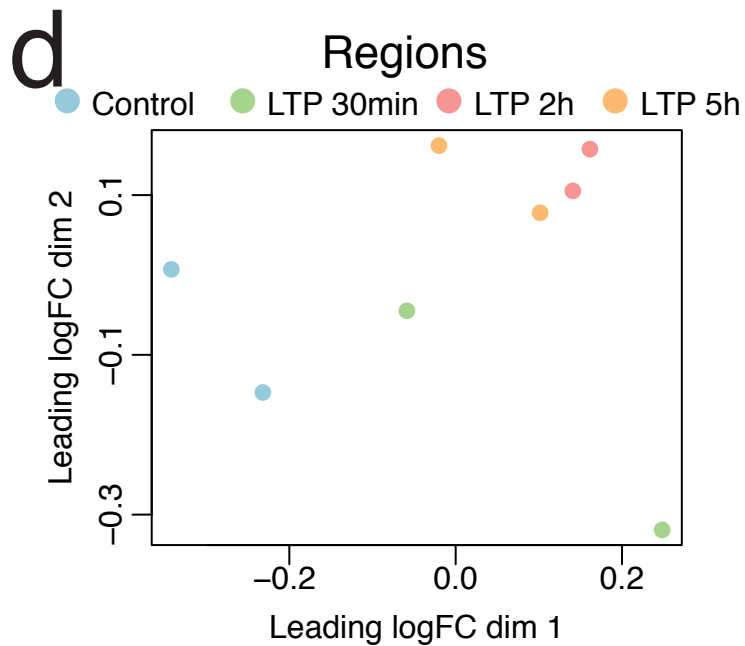

Supplement: Supplementary file 9 — Group post-synaptic potential changes in response to HFS, and average array intensity and sample clustering. (a) The differences in the groups’ field excitatory post-synaptic potential after HFS. The data was taken from Maag et al. 2015, and included all 3 samples per group. The control group in this graph is the baseline test stimuli used as control in the present study (b) Distribution of probe intensity per samples before quantilie normalisation. MDS plot of (c) probes and (d) regions after quantile normalisation. (PDF 1131 kb) [file 12864_2017_3621_MOESM9_ESM.pdf]
