# Supplementary material for: Widespread promoter methylation of synaptic plasticity genes in long-term potentiation in the adult brain in vivo
Source: BMC Genomics. 2017 Mar 23;18:250. doi: 10.1186/s12864-017-3621-x (PMC5364592; doi:10.1186/s12864-017-3621-x)
Supplement: Supplementary file 4 — Correlation between expression and methylation changes after HFS. Gene log fold changes of DE genes were imported from Maag et al. 2015. For each time point, all DE genes logFC were plotted against the logFC methylation after HFS vs. control. Each gene has multiple corresponding methylation regions based on figure 1b. Lines were plotted at ± 0.9 logFC expression and ± 0.1 logFC methylation. Each resulting quadrant shows the number of genes present. (PDF 118 kb) [file 12864_2017_3621_MOESM4_ESM.pdf]

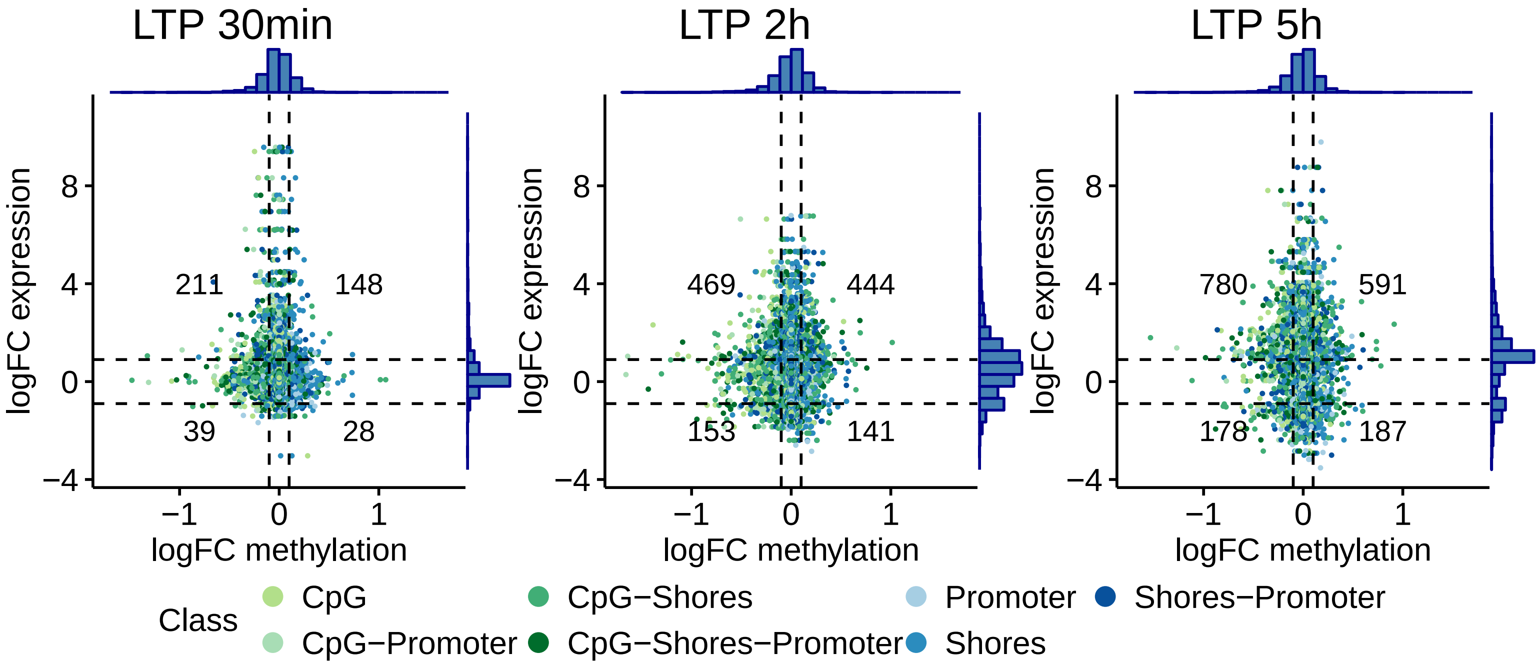
LTP 30min
LTP 2h
LTP 5h
logFC expression
logFC methylation
8
4
0
−4
−1
0
1
211
148
39
28
469
444
153
141
780
591
178
187
Class
CpG
CpG−Promoter
CpG−Shores
CpG−Shores−Promoter
Promoter
Shores
Shores−Promoter
