# Supplementary material for: Widespread promoter methylation of synaptic plasticity genes in long-term potentiation in the adult brain in vivo
Source: BMC Genomics. 2017 Mar 23;18:250. doi: 10.1186/s12864-017-3621-x (PMC5364592; doi:10.1186/s12864-017-3621-x)
Supplement: Supplementary file 7 — Investigation of GC-content in differentially methylated probes and regions reveals no bias between differential methylation and GC-content. GC-content of each (a) probe and each (b) region compared to the t-statistics from limma when comparing to control. (c) GC-content of each region compared to the correlation value when compared to gene expression. (PDF 2187 kb) [file 12864_2017_3621_MOESM7_ESM.pdf]

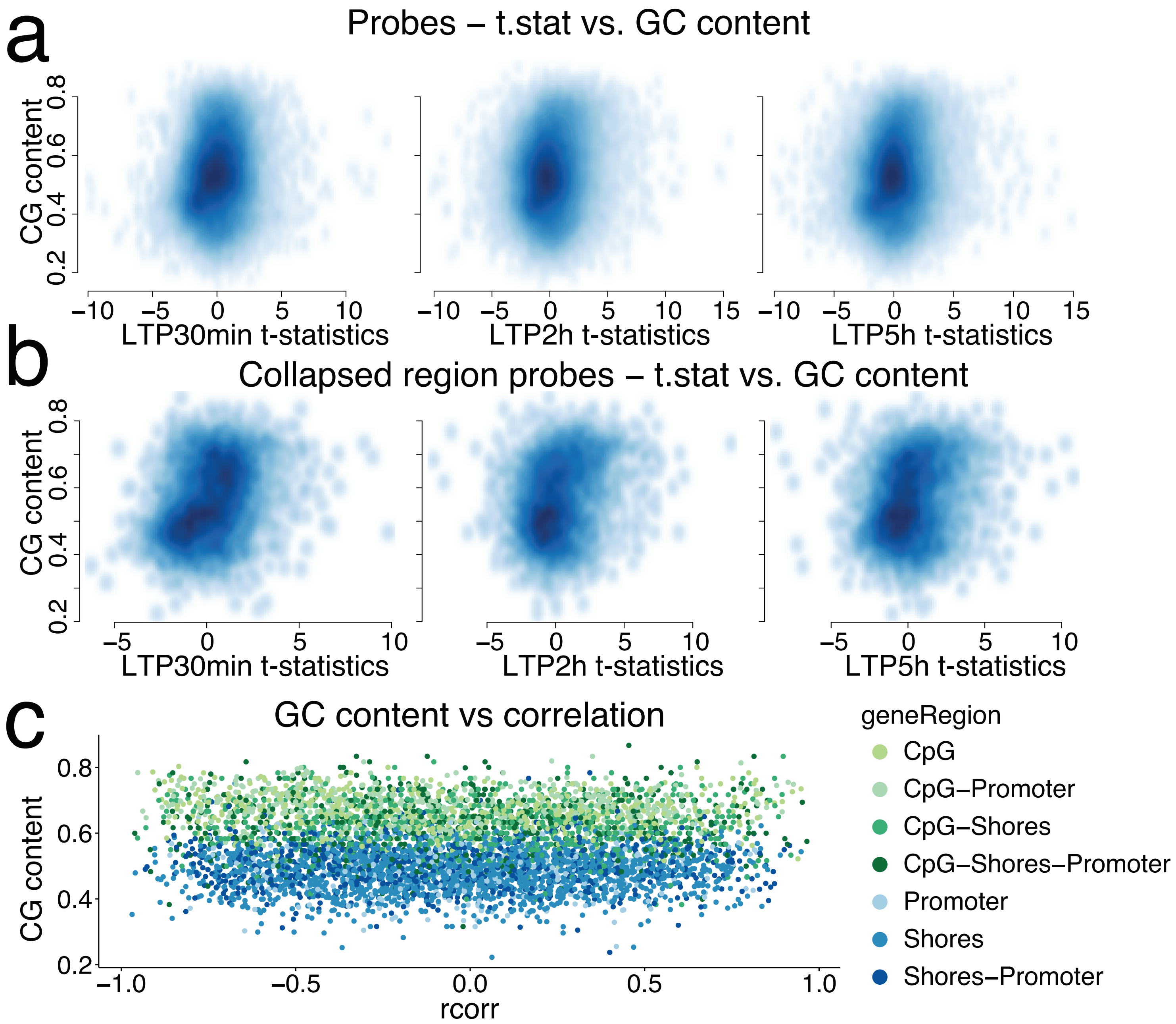
a
Probes – t.stat vs. GC content
CG content
0.2
0.4
0.6
0.8
−10
−5
0
5
10
15
LTP30min t-statistics
LTP2h t-statistics
LTP5h t-statistics
b
Collapsed region probes – t.stat vs. GC content
CG content
LTP30min t-statistics
LTP2h t-statistics
LTP5h t-statistics
c
GC content vs correlation
CG content
rcorr
−1.0
−0.5
0.0
0.5
1.0
geneRegion
CpG
CpG−Promoter
CpG−Shores
CpG−Shores−Promoter
Promoter
Shores
Shores−Promoter
